# Origin of Some Nursery Rhymes

**Published:** 1884-07

**Authors:** 


### ORIGIN OF SOME NURSERY RHYMES.

"CINDERELLA; or, the Glass Slipper," is a very old story. Thousands of years ago it was told to boys and girls. The original read that an eagle stole the slipper of a very pretty Egyptian lady and bore it off. The eagle dropped it, and some one carried it to the king, who made it known all over his kingdom that he would marry the lady whose tiny foot it should fit. And so Cinderella, the cinder-girl, became queen. In France, Germany, and this country the story has taken several shapes, and has always been a favorite.

"Little Jack Horner" has the following history: In England, in the reign of Henry, VIII., there lived a Mr. Horner. Henry, the King, wished to tear down all the fine monasteries and abbeys of England, sell their lands and pocket the money. This Mr. Horner was butler, or something, to an old abbot, who thought he would gain favor with Henry by giving him twelve of his very best and richest monasteries. So the abbot sent deeds of them to the king by this John Horner. But Horner thought as he "sat in the corner" of the carriage on his way to the king, that he would see what all those great papers which he was carrying should mean. "He put in his thumb and pulled out a plum"—*i. e.*, he opened and read the deeds, put the one for the largest piece of land in his own pocket, gave the rest to the king at London, and came home and told his master that Henry VIII., for his fidelity, had made him a present of one of the large tracts of land.

"Blue Beard," too, is very old. He is supposed to be Giles Delaval, Lord of Rais, and was Marshal of France in 1429.

"Jack, the Giant-killer," came from India. He breaks forth in all sorts of doings all over the story-books of the young. And so of "Jack and the Bean-stalk."

"Babes in the Wood" is a very touching story. I think the origin of this may be considered a very, very old ballad, which tells of Richard III, murdering his own dear little nephews.

"The story of "Little Red Riding-Hood" is found in the German, but not exactly as we tell it in English.

The Germans have a great variety of young-folk-lore, or stories for little ones.

"Mother Goose" was a real person. She lived in Boston. Her daughter Elizabeth married the printer, Tom Fleet, who gathered up the nursery melodies of his mother-in-law and published them.

I must now tell you of the meaning of an old nursery rhyme: "Four and twenty black-birds made into a pie:" these are the four and twenty hours of the day; the "pie" is the space between the earth and the sky, the flat-looking ground being the bottom crust, the birds in between, and the sky being the concave top crust.

"When the pie was opened," *i. e.*, when day began to break. "The birds began to sing," *i. e.*, the hours to begin merrily. "The king was in the parlor counting out money;" the "king" is the sun, the monarch of the day. There he is enthroned in the sky. He is said to be counting out money, because the sunshine is gold-color. See how he "counts it out," flings it about him, the beautiful golden sunshine. "The queen upstairs eating bread and honey." Of course, if the king is the sun, the queen is the moon. "The maid in the garden, hanging out clothes." This "maid" is Aurora, the goddess, not of the day, but of the dawn. Now, "up jumped a little bird and nipped off her nose." The little bird who did this very ungallant thing is, of course, the first hour of the day, for Aurora, or dawn, disappears as soon as the king, or sun, arises.

---

In those forms of neuralgia and rheumatism which have a malarial origin—and most seem to arise from such causes—the action of "Tongaline" in connection with quinine has been found very satisfactory, the therapeutic properties of both seeming to be accentuated under the circumstances. With each dose of "Tongaline" two to five grains of quinine is prescribed, according to the severity of the attack and the susceptibility of the patient to the effects of the latter.—[Extract from the July No. of the "Medical Brief."

